# Association Between Teprotumumab and Hearing Impairment: A Meta-Analysis

**DOI:** 10.7759/cureus.102775

**Published:** 2026-02-01

**Authors:** Maxim J Barnett, Maria deMelo, Maria Rego, Carlo Casipit

**Affiliations:** 1 Internal Medicine, Jefferson Einstein Philadelphia Hospital, Philadelphia, USA

**Keywords:** exophthalmos, graves' orbitopathy, tepezza, teprotumumab, thyroid eye disease

## Abstract

Teprotumumab is a medication for thyroid eye disease. We conducted a review of studies assessing teprotumumab for thyroid eye disease treatment from MEDLINE (Medical Literature Analysis and Retrieval System Online) and CINHAL (Cumulative Index to Nursing and Allied Health Literature) from inception to September 20, 2025. The outcome of interest was hearing impairment. We included nine studies (four randomized controlled trials and five observational studies). The randomized controlled trials included 153 teprotumumab-treated patients. Using both broad and strict definitions of hearing loss, teprotumumab continued to demonstrate a statistically significant increased risk of hearing impairment (Broad: risk ratio (RR) 3.57, 95% CI 1.27-9.97; Strict: RR 5.23, 95% CI 1.40-19.57). Observational studies included over 2,000 patients treated with teprotumumab. Using the broad definition, the RR amongst observational studies was 2.78 (95% CI, 2.38-3.24); using the strict definition, the RR amongst observational studies was 2.76 (95% CI, 2.30-3.31). Sensitivity analyses were performed, with results remaining statistically significant, without heterogeneity or publication bias. Teprotumumab is associated with hearing impairment using broad and strict definitions, consistent amongst both RCTs and observational studies. Further research is required to address potential treatment options, the likelihood of recurrence with retreatment, and the chance of recovery.

## Introduction and background

In 1835, Robert Graves first used the term ‘exophthalmic goiter’; unbeknownst to him at the time, this had been documented (posthumously) by Caleb Perry one decade earlier [1]. Over the ensuing century, the term has become interchangeable with Graves’ orbitopathy (ophthalmopathy) and thyroid eye disease (TED). Around 50% of all patients with Graves’ disease have TED; however, up to 90% of patients with TED have Graves’ disease [2]. In patients with Graves’ disease, TED most commonly occurs within one year of diagnosis; however, in up to 15% of patients, TED may precede the diagnosis of Graves’ disease [3].

The psychosocial impacts of TED are often underappreciated. Nearly 65% of patients with moderate-to-severe impairment reported an impaired quality of life [4]. Additionally, up to 45% of patients report constant limitations in activities of daily living [4]. Anxiety and depression are evident in 45% of patients, and around 50% of all patients report poor self-confidence [4]. Furthermore, one in five patients avoids public situations [4].

In January 2020, teprotumumab became the first medication to be approved by the United States Food and Drug Administration (FDA) for TED. Teprotumumab (brand name: Tepezza) is a monoclonal antibody that inhibits the insulin-like growth factor-1 receptor (IGF-1R), preventing the cross-talk with thyroid-stimulating hormone (TSH) receptors, disrupting fibroblast activation and inflammatory cytokine production. It is approved for TED of any disease activity and duration. Since early clinical studies of this medication, adverse outcomes, including otologic impairment, have been reported, leading the FDA to require a label warning of “hearing impairment” in July 2023 [5].

At present, the potential mechanism of hearing loss (and other adverse otologic events) in relation to teprotumumab remains incompletely understood, as well as the likelihood for recovery, methods of prevention, and treatment options. This study aims to analyze the available literature and review the proposed mechanism, pathophysiology, and treatment recommendations, in addition to performing a meta-analysis to provide an overall estimate of effect for the adverse otologic event of hearing impairment.

## Review

Methodology

Search Strategy

Both MEDLINE (Medical Literature Analysis and Retrieval System Online) and CINHAL (Cumulative Index to Nursing and Allied Health Literature) platforms were used for the literature search, from inception to September 2025. Search string included “teprotumumab”, “tepezza”, “hearing impairment”, “hearing loss”, “tinnitus”, “autophony”, “hyperacusis”, “eustachian tube dysfunction” or “patulous eustachian tube.” No restrictions were implemented in relation to race, gender, age, language, or geographic region. The Preferred Reporting Items for Systematic reviews and Meta-Analyses (PRISMA) guidelines were followed for this review [6]. 

Eligibility Criteria

Studies were considered eligible if they (i) were observational studies (case-control or cohort), (ii) randomized controlled trial (RCTs) (or non-RCTs), (iii) evaluated teprotumumab in TED compared to another medication or placebo, (iv) provided an odds ratio (OR), risk ratio (RR) or hazard ratio (HR) (as well as any variance measure such as 95% confidence intervals (CIs)) for between-group comparisons. Included studies furthermore involved adult patients with a diagnosis of Graves’ ophthalmopathy (irrespective of severity or duration), with an experimental group (monotherapy with teprotumumab) and a control group (placebo or standard therapy).

Studies were deemed ineligible if they were other publication types (such as case reports or background articles), provided insufficient data, or involved non-human studies.

References from all included studies, prior systematic reviews, and meta-analyses were also searched manually for additional eligible studies. The outcome analyzed was hearing impairment.

Statistical Analysis

A meta-analysis was done of RRs on the log scale. When studies reported ORs or HRs, these were analyzed as RRs due to low incidences, and the exact baseline risk was unknown. A random-effects model was incorporated due to the heterogeneous protocols of the studies and likely background populations. Standard errors and point estimates were calculated using DerSimonian and Laird’s generic inverse variance method. Heterogeneity was assessed using Cochrane’s Q test with I^2^ supplementation; if Q was < 0.05, heterogeneity was assumed to be present. I^2^ was defined as insignificant (0-25%), low (26-50%), moderate (51-75%), and high (above 75%). A forest plot was separately performed for RCTs and observational studies. Statistical analyses were performed through an online platform [7]. Outcomes included hearing impairment (using a broad definition, encompassing sensorineural and conductive hearing loss, tinnitus, eustachian tube dysfunction, and patulous eustachian tube), and a strict definition of hearing impairment (hearing loss/hypoacusis).

Risk of Bias Assessment

RCTs utilized the Robvis risk of bias tool [8]; conversely, observational studies were analyzed with the Newcastle Ottawa Scale (NOS) [9] to determine the accuracy of outcomes, quality of recruitment, and comparability between groups (with a score of greater than seven consistent with high-quality studies).

Results

**Figure 1:**
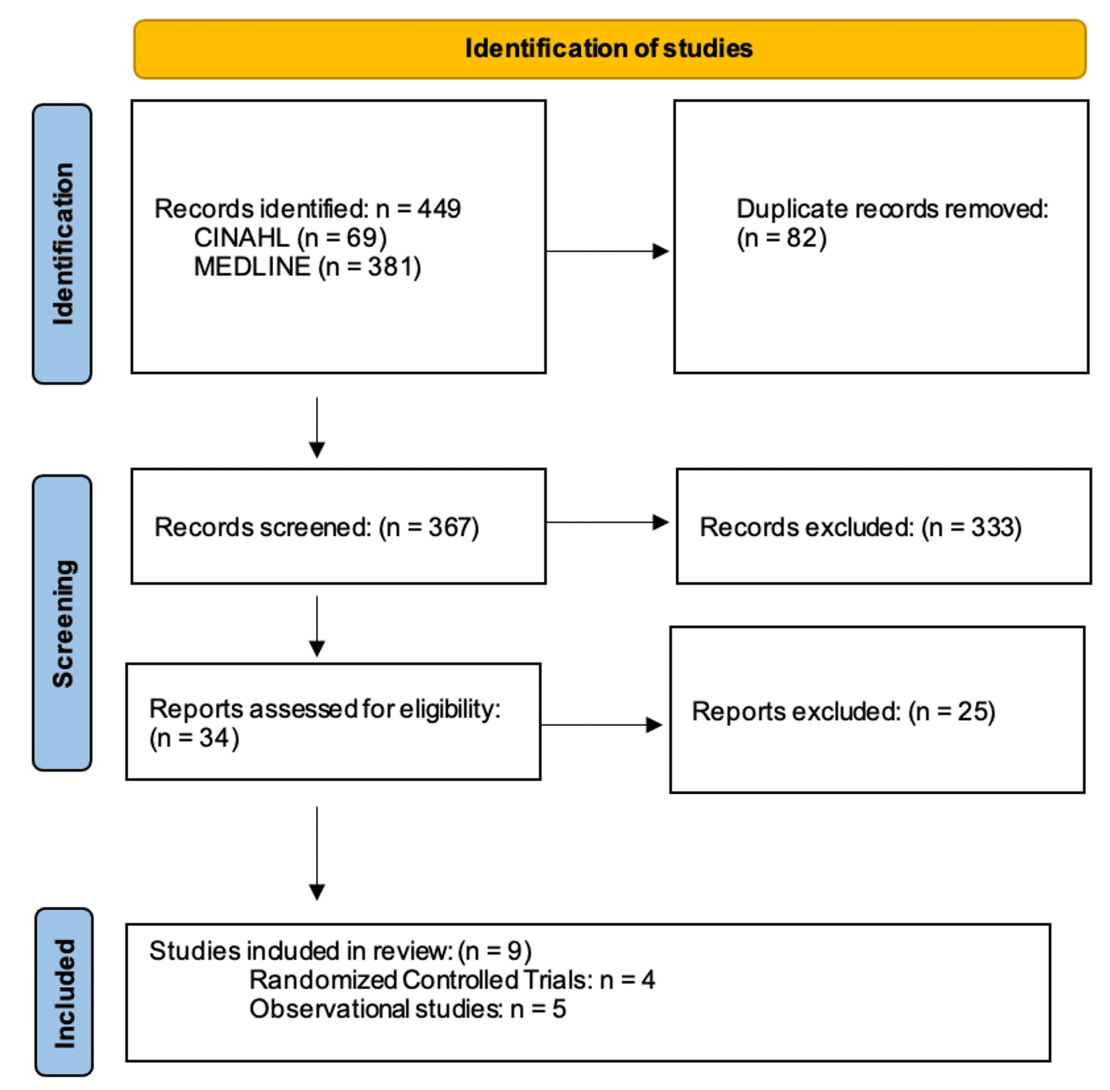
PRISMA flow chart of selection of studies PRISMA: Preferred Reporting Items for Systematic reviews and Meta-Analyses

The initial search strategy identified 381 studies from MEDLINE and 69 from CINHAHL. After removing duplicates (n = 82), a total of 367 were analyzed. Of these, 333 did not meet eligibility criteria. An additional 25 were excluded after screening the title and abstract, leading to nine (four RCTs [10-13] and five observational studies [5,14-17]). Figure 1 shows the PRISMA flowchart, and Table 1 gives the summary of the included studies.

**Table 1:** Characteristics of randomized controlled trials and observational studies CI: confidence interval; IV: intravenous; N/A: not applicable; PO: *per os* (orally); RR: relative risk

Author(s)	Year	Country	Study Design	Number of Participants	Hearing Impairment Definition	Outcome (Broad Definition)	Outcome (Strict Definition)
Barnett et al. [5]	2025	United States	Retrospective Cohort (Multicenter)	Teprotumumab: 532, Placebo: 532	Sensorineural hearing loss	N/A	RR = 3.21 (95% CI 1.936-5.323)
Smith et al. [10]	2017	United States and European Union	Randomized Controlled Trial (Multicenter)	Teprotumumab: 43, Placebo: 44	Hearing impairment and tinnitus	RR = 7.16 (95% CI 0.38-134.6)	RR = 5.11 (95% CI 0.25-103.52)
Douglas et al. [11]	2020	United States and European Union	Randomized Controlled Trial (Multicenter)	Teprotumumab: 41, Placebo: 42	Hypoacusis, deafness, autophony, patulous eustachian tube	RR = 11.26 (95% CI 0.64-197.38)	RR =7.17 (95% CI 0.38-134.56)
Douglas et al. [12]	2023	United States	Randomized Controlled Trial (Multicenter)	Teprotumumab: 42, Placebo: 20	Eustachian tube dysfunction, tympanic membrane disorder, tinnitus, hypoacusis, conductive deafness, unilateral deafness	RR = 2.14 (95% CI 0.51-9.01)	RR = 6.35 (95% CI 0.38-107.45)
Hiromatsu et al. [13]	2025	Japan	Randomized Controlled Trial (Multicenter)	Teprotumumab: 27, Placebo: 27	Patulous eustachian tube, neurosensory hypoacusis, tinnitus	RR = 4 (95% CI 0.48-33.51)	RR = 4 (95% CI 0.48-33.51)
Toro-Tobon et al. [14]	2023	United States	Retrospective Case-Control (Multicenter)	Teprotumumab: 18, Tocilizumab: 6	Hearing loss, tinnitus, ear fullness, non-hearing loss symptoms	RR = 3.32 (95% CI 0.20-54)	RR = 2.58 (95% CI 0.15-43.86)
Markle et al. [15]	2025	United States	Retrospective Cohort (Multicenter)	Teprotumumab: 441, Placebo: 443	Tinnitus, sensorineural hearing loss, hypoacusis, hyperacusis, autophony, eustachian tube dysfunction	RR = 2.85 (95% CI 1.94-4.20)	N/A
Hori et al. [16]	2025	United States	Retrospective Cohort (Multicenter)	Teprotumumab: 947, Placebo: 947	Sensorineural hearing loss and tinnitus	RR = 2.85 (95% CI 2.10-3.85)	RR = 2.89 (95% CI 1.92-4.36)
Lo et al. [17]	2025	United States	Retrospective Cohort (Multicenter)	Teprotumumab: 923, IV Corticosteroid: 685, PO Corticosteroid: 741, Conservative: 892	Conductive and sensorineural hearing loss, requirement for hearing device	Teprotumumab vs intravenous corticosteroids: RR = 2.43 (95% CI 1.67-3.55); Teprotumumab vs oral corticosteroids: RR = 2.38 (95% CI 1.65-3.44); Teprotumumab vs conservative therapy: RR = 3.298 (95% CI 2.202-4.941)	N/A

Risk of Bias Analysis 

**Figure 2:**
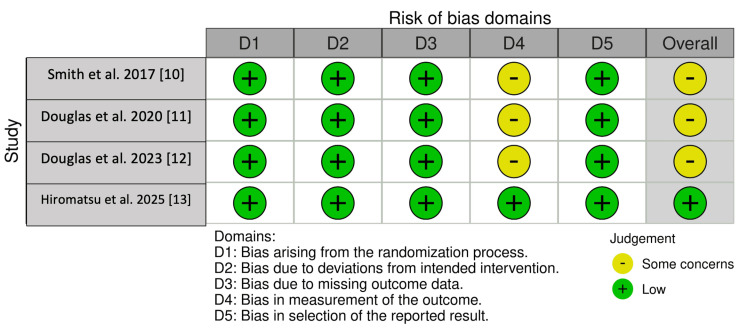
Risk of bias for included randomized controlled trials

Risk of bias assessment of the RCTs demonstrated a low risk across several domains using the Robvis tool, with some concerns identified in select studies, particularly in relation to deviations from intended interventions and outcome measurement (Figure 2). No RCT was assessed as having a high risk of bias across multiple domains. Observational studies were rated as moderate-to-high quality based on NOS scores (ranging from 7-9), reflecting generally adequate selection and outcome assessment; however, inherent limitations to observational designs, such as potential for residual confounding and misclassification, cannot be excluded (Table 2). Overall, these findings suggest that while the included studies are subject to methodological limitations, the risk of bias was acceptable for inclusion in the present meta-analysis. 

**Table 2:** Risk of bias assessment for included observational studies

Study	Year Published	Newcastle-Ottawa Scale	Selection	Comparability	Outcome
Toro-Tobon et al. [14]	2023	Overall: 7	2	2	3
Markle et al. [15]	2024	Overall: 9	4	2	3
Barnett et al. [5]	2025	Overall: 8	3	2	3
Hori et al. [16]	2025	Overall: 9	4	2	3
Lo et al. [17]	2025	Overall: 9	4	2	3

Randomized Controlled Trials

**Figure 3:**
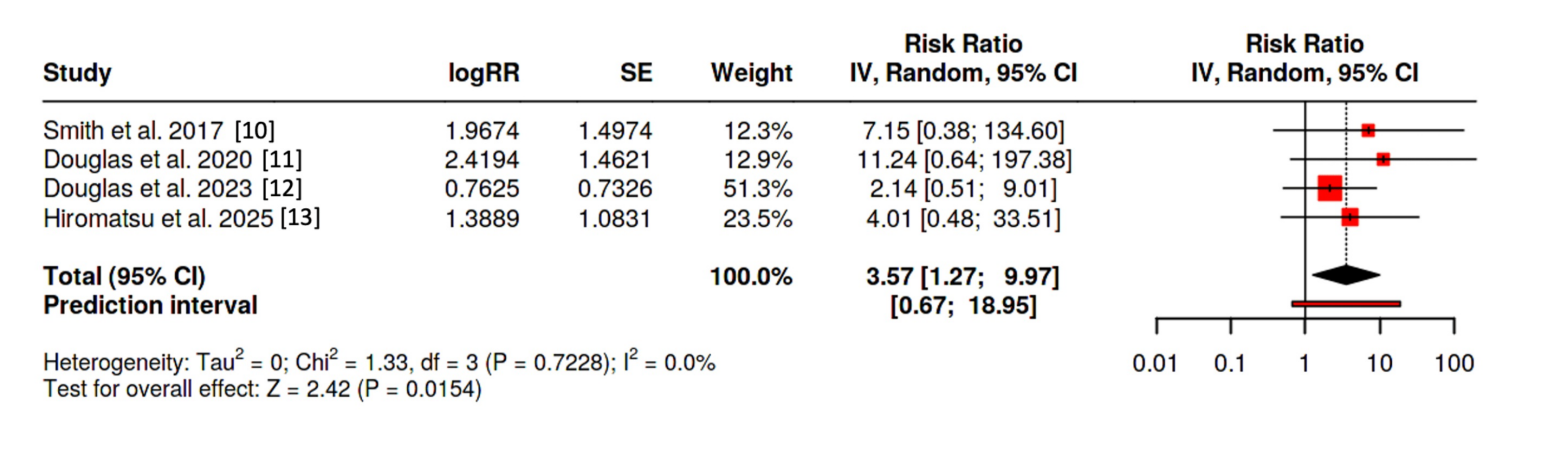
Forest plot of randomized controlled trials using “broad” definition

An analysis composed solely of RCTs [10-13] was performed. As solely hypoacusis was reported among the trial posed by Hiromatsu et al. (fulfilling both the broad and strict definitions) [13], the same outcome was used for both estimates. With the broad definition of hearing impairment, the pooled analysis demonstrated a relative risk of 3.57 (95% CI: 1.27-9.97, p = 0.0154). Heterogeneity was not present (I^2^ = 0) in our study (Figure 3). Egger’s test supported the presence of asymmetry (p = 0.024); therefore, using a “Trim-and-Fill” analysis, our results remained statistically significant (RR 2.61; 95% CI 1.04-6.54, p = 0.0409).

**Figure 4:**
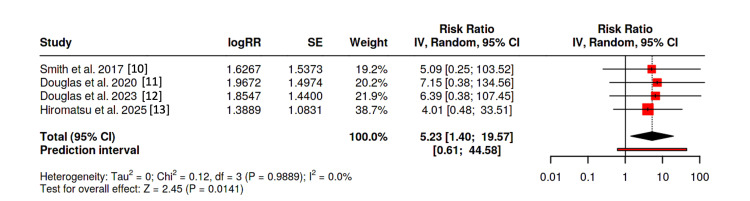
Forest plot of randomized controlled trials using “strict” definition

Using the strict definition of hearing loss, the pooled analysis demonstrated a relative risk of 5.23 (95% CI: 1.40-19.57, p = 0.0141). Heterogeneity again was absent (I^2^ = 0) in our study, and Egger’s test failed to demonstrate asymmetry (p = 0.163) (Figure 4).

Observational Studies

As the study by Barnett and colleagues solely analyzed sensorineural hearing loss, the same outcome was used for both the broad and strict definitions [5]. Additionally, the study by Markle et al. was reported as OR, which was analyzed in our forest plot as a relative risk [15]. Lo et al. similarly provide HRs, which were included in our forest plot as RRs. Additionally, they analyzed solely hearing loss as the outcome, fulfilling both the broad and strict definitions with the same estimate [17]. On the other hand, Hori et al. provided two separate outcomes of sensorineural hearing loss and tinnitus, and we estimated an RR fulfilling the broad definition; we used a sensitivity analysis by varying the likely prevalence of overlap between tinnitus and hearing loss, remaining statistically significant [16].

**Figure 5:**
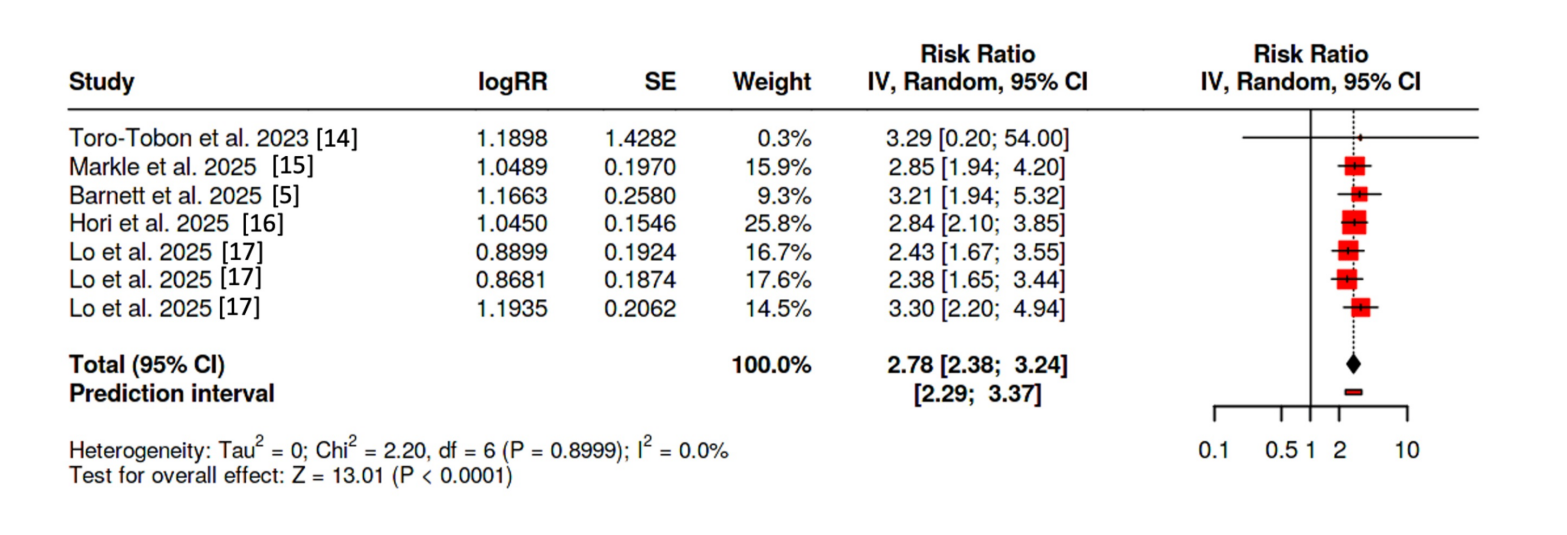
Forest plot of observational studies using “broad” definition

Using the broad definition of hearing impairment, five studies [5,14-17] were included for an overall estimate. The pooled analysis demonstrated an RR of 2.78 (95% CI 2.38-3.24, p < 0.0001), without heterogeneity (I^2^ = 0) (Figure 5); Egger’s test failed to demonstrate asymmetry. Exclusion of the studies by Toro-Tobon et al. (comparing teprotumumab to tocilizumab) [14], as well as two of the three arms of Lo et al. were performed (oral/intravenous corticosteroids to teprotumumab) [17]; however, the results remained statistically significant (RR 2.99; 95% CI 2.47-3.62, p < 0.0001).

**Figure 6:**
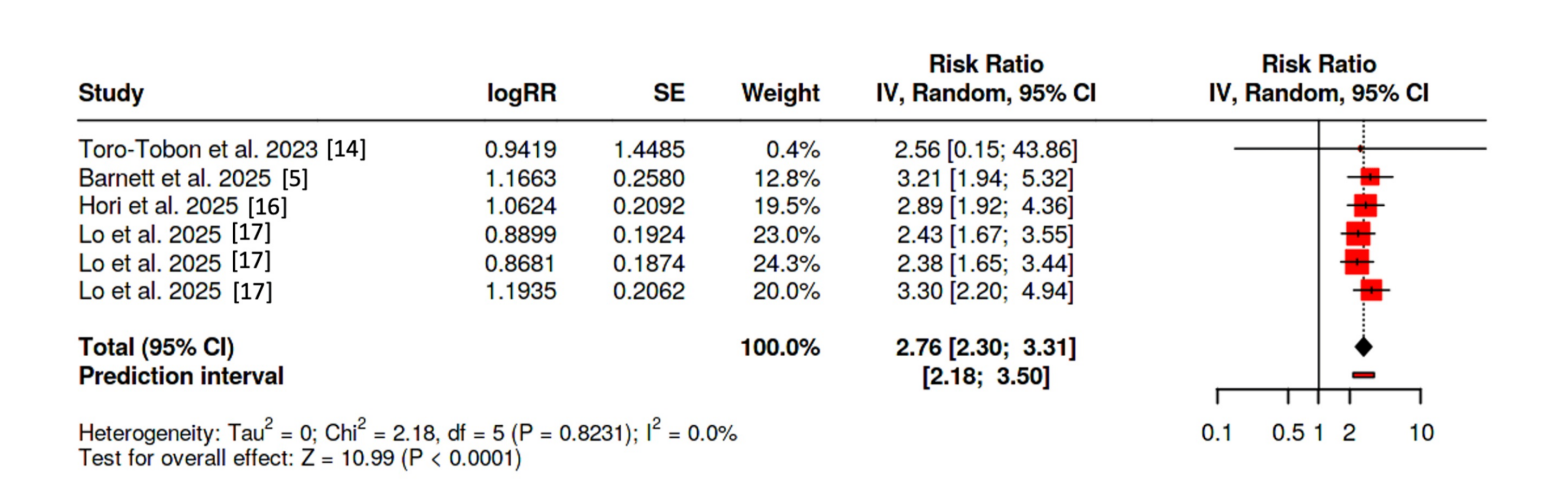
Forest plot of observational studies using “strict” definition

Using the strict definition of hearing impairment, four studies were included (the study by Markle and colleagues [15] was excluded as a strict definition was not possible with the data provided). The pooled relative risk was 2.76 (95% CI 2.30-3.31, p < 0.0001) without heterogeneity (I^2^ = 0); furthermore, Egger’s test failed to demonstrate asymmetry (p = 0.77) (Figure 6). The study by Toro-Tobon and colleagues [14] and two of the three arms of Lo et al. [17] were again removed, with the results remaining statistically significant (RR = 2.89; 95% CI 2.35-3.56, p < 0.0001).

Discussion

To the best of our knowledge, this is the first meta-analysis to solely assess the effects of teprotumumab on hearing impairment. Our report included nine studies, comprised of four RCTs and five observational studies. The RCTs included 153 teprotumumab-treated patients, and the observational studies included over 2,000 patients treated with this medication. Our meta-analysis was performed based on the differing definitions of the studies, which provided a “broad definition” (encompassing all otic changes such as hearing loss, tinnitus, aural fullness, autophony, hyper/hypoacusis, and eustachian tube dysfunction, amongst others) and a “strict definition” (solely hearing loss). Both RCTs and observational studies (with both definitions) consistently demonstrated a statistically significant increased risk for adverse hearing events, irrespective of the definition used. 

The initial incidences of adverse otologic events from randomized controlled trials were reported at around 10%; however, observational studies demonstrated a significantly greater prevalence than previously anticipated [10,11]. For example, Sears et al. demonstrated that 22 participants (of 27) noted subjective otologic symptoms [18]. Throughout the various studies, a particular fallacy was the definition of “hearing impairment,” which could vary from sensorineural hearing loss, hypoacusis, autophony, tinnitus, ear fullness, patulous eustachian tube, and/or hyperacusis. Sensorineural hearing loss appears to be the most common and perhaps the greatest concern. Notable risk factors documented within the literature include elderly age, concurrent ototoxic medications, pre-existing hearing impairment, higher clinical activity score, and loud noise exposure [18]. Key et al. state the most common timing for symptom onset is between infusions three and five (median 3.8 infusions) (weeks six through 12); however, the authors demonstrate a wide range reported within the literature, ranging from three to 37 weeks after initiation of teprotumumab [19]. Interestingly, Toro-Tobon et al. suggest that, although there is a greater risk of hearing impairment with teprotumumab compared to corticosteroids, there is no statistically significant difference in the requirement of hearing devices [14].

There are wide discrepancies in the reported incidence of hearing impairment following teprotumumab. As noted by Epperson et al., just over 50% of all patients exposed to teprotumumab who experienced subjective hearing impairment reported it; for those who did not, the patients were either unaware of the connection with the medication or were unaware of how to report it [20]. Wong et al. note that around half of all patients with hearing loss (identified with audiometry) do not report any symptoms, with another one-third of patients reporting otologic symptoms without hearing loss [21]. Yu et al. remind us that subjective and objective hearing loss are in concordance less than 72% of the time [22]. Wong et al. hypothesize that this discordance may be due to frequencies above 8 kHz (ultrahigh frequencies), which may be the first manifestation, and are typically outside of the range of testing on audiometry (the authors furthermore state medication-related ototoxicity is known to manifest at ultrahigh frequencies) [21].

Pharmacology

Teprotumumab is a fully human IgG1 monoclonal antibody that antagonizes the IGF-1 receptor and is the only FDA-approved medication for TED [5]. Teprotumumab is administered as an intravenous infusion over 90 minutes, and its course requires six months of treatment. The initial dose is 10 mg/kg, followed by 20 mg/kg every three weeks (over 24 weeks) to complete eight infusions [23]. It has a small volume of distribution, low systemic clearance, and a long half-life of 20 days [23]. Teprotumumab exhibits linear kinetics, and its metabolism is believed to occur with proteolysis into small amino acids and peptides (rather than cytochrome enzymatic degradation or renal excretion) [23]. Presently, the American Thyroid Association (ATA) and the European Thyroid Association (ETA) estimate that a full treatment of teprotumumab will cost nearly $360,000 USD [24]. By 2024, an estimated 14,500 patients have received treatment with Teprotumumab [25].

Teprotumumab was initially developed in 2008 for investigation into the management of oncological diseases; however, it never received approval for this purpose [5]. Although a breakthrough in TED management, teprotumumab is not without adverse effects (such as muscle cramps, alopecia, hyperglycemia, inflammatory bowel disease flare, and hearing impairment) [24]. Intriguingly, hearing changes were not documented in these initial oncological trials [26]. Hearing impairment is likely to be the most alarming symptom. In July 2023, the FDA added a label warning of hearing impairment; as a result, this led to a drop in sales and litigation against Horizon [5,27]. Nearly 88% of patients who experience hearing impairment with teprotumumab note an impaired quality of life, and 33% expressed regret taking the medication [20].

Other IGF-1 receptor antagonists are currently under investigation, which have assisted in furthering the understanding of hearing impairment as an adverse event. Veligrotug (VRDN-001), developed by Viridan Therapeutics (Waltham, Massachusetts, United States), is another humanized monoclonal antibody inhibiting IGF-1 receptor; it is administered as 10 mg/kg for a total of five infusions (over 30 minutes) every three weeks with a half-life of 10 days, and has demonstrated comparable rates of hearing loss with teprotumumab. Linsitinib, an oral IGF-1 and insulin receptor antagonist developed by Astellas Pharma Inc. (Tokyo, Japan) (in-licensed to Sling Therapeutics, Ann Arbor, Michigan, United States) has demonstrated efficacy in TED without any documentation of hearing impairment [28].

Recovery

The documented rates of recovery of hearing loss are variable throughout the literature. Fallacies include failure to follow up patients long-term and failure to provide audiometric confirmation of recovery (or persistence of impairment). Similarly, the recovery differs based on the specific hearing complication. Overall, Sears et al. estimate that around 50% of patients (when using the broad definition of hearing impairment) will have remission upon treatment discontinuation; this appears most likely with tinnitus (100%), followed by ear fullness (91%), autophony (83%), with sensorineural hearing loss (hypoacusis) the least likely to resolve (46%) [18].

Re-Exposure to Teprotumumab

A particular area of uncertainty is the risk of recurrence of TED following completion of therapy. It is estimated that around 40% of patients will develop a relapse of proptosis, most commonly within the first year [24]. While teprotumumab may be re-initiated, a particular caveat, however, is the risk for recurrent (and worsening) hearing impairment upon re-exposure. As demonstrated in the OPTIC-X study, patients with an initial episode of hearing impairment are not immune to repeat episodes upon re-exposure [29].

Proposed Pathophysiology

**Figure 7:**
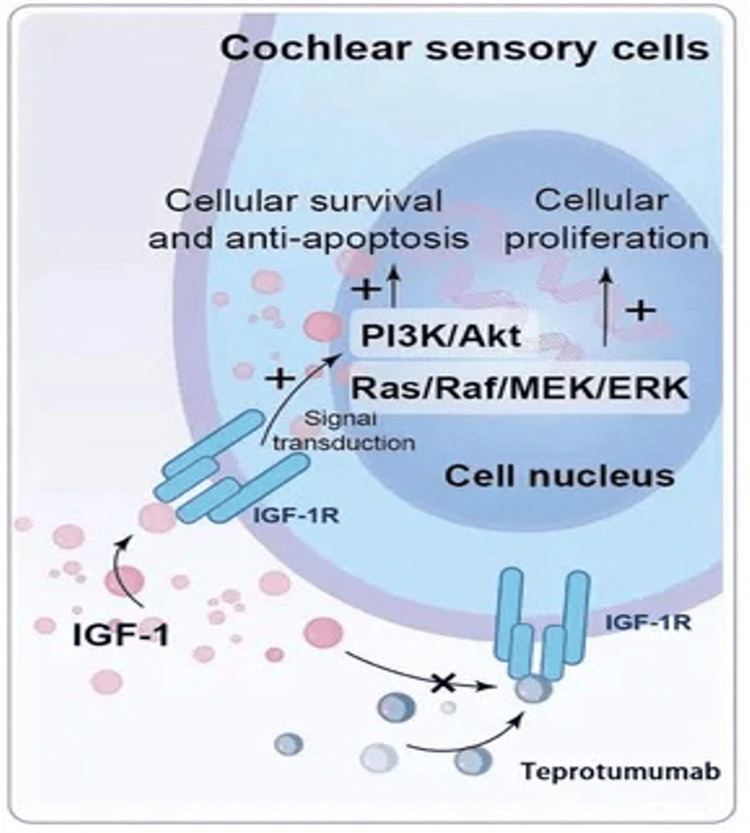
Cochlear sensory cells and mechanism of action of teprotumumab Image Source: Xu et al., 2025 [30]; Published under the terms of CC BY 4.0: Attribution 4.0 International Deed

Under normal circumstances, IGF-1 exerts neurotrophic mechanisms on cochlear hair cells and synapses. IGF-1 binds to cochlear sensory cells, leading to intracellular activation of signalling pathways (including PI3K/Akt and Ras/Raf/MEK/ERK), leading to expression of Netrin1 and Gap43, promoting survival, maintenance, and proliferation (alongside inhibition of apoptosis) [26]. Teprotumumab binds to the IGF-1 receptor, disrupting this interaction, leading to an inhibition of intracellular signalling, with damage and reduction of cochlear sensory and glial cells (Figure 7) [30]. IGF-1 receptor knockout mice demonstrate improper hair cell development [31]. Reed et al. suggest it may not be the direct ototoxic effects of Teprotumumab, but rather the increased susceptibility to noise-induced trauma (coupled with inability to recover); the authors pose a case of acute hearing impairment in a patient treated with teprotumumab following exposure to a rifle blast [32].

Treatment of Ototoxicity

At present, there is no treatment for teprotumumab-related hearing loss, apart from cessation of the medication. Traditionally, idiopathic sensorineural hearing loss has been treated with corticosteroid administration [33]. The usage of corticosteroid therapy in teprotumumab-associated hearing loss, however, is poorly defined. In normal physiology, IGF-1 promotes inflammation in normal physiology, and therefore inhibition is unlikely to lead to inflammation within the cochlea [33]. While some reports fail to demonstrate any benefit from oral corticosteroid therapy following teprotumumab exposure, others, such as the case posed by Lu et al. (who initiated corticosteroids within 48 hours of hearing impairment), noted improved outcomes [34]. Notably, the authors state the patient was able to continue teprotumumab without further hearing impairment, suggesting corticosteroids may provide ongoing protective mechanisms.

The optimal dose, duration, and frequency of teprotumumab is not completely defined; the recommendation of 20 mg/kg every three weeks comes from a Roche model suggesting above 20 μ/ml is required for greater than 90% IGF-1 receptor saturation [35]. It should be noted, however, that preliminary studies (for oncological purposes) trialled differing dosages, such as 9 mg/kg weekly and 16 mg/kg every three weeks, without reports of hearing loss [35]. This would suggest less-frequent dosing, or overall dosages would be beneficial. The study by Ho et al. occurred during the SARS-CoV-2 pandemic, whereby production of teprotumumab was interrupted; as a result, patients received a mean of 4.2 infusions, with statistically significant reductions in TED; however, they continued to demonstrate hearing impairment (which was persistent) [36]. Lustig-Barzelay et al. furthermore note improvement in ophthalmopathy in more than 40% of patients after two to three infusions [37]. Phansalkar et al. present a patient receiving teprotumumab requiring discontinuation after four dosages due to hearing impairment; the patient, however, restarted at half-dosage (10 mg/kg) and was able to complete eight infusions without objective worsening [35]. With monoclonal antibodies, the time above the inhibitory threshold is virtually 100%; however, when considering linsitinib (twice-daily dosing), fluctuations in serum levels from its short half-life may allow for recovery of cochlear IGF-1 receptors in between.

Certain diseases, such as Laron-Type dwarfism, predispose to sensorineural hearing loss; recombinant IGF-1 administration is known to prevent (and improve existing) hearing loss in these patients [38]. Another consideration is age-related hearing loss, which is correlated with a decline in IGF-1 levels; age-related hearing loss (presbycusis) is characterized by impairment of high frequencies, which is the pattern also noted with teprotumumab therapy [18,39]. In animal studies, recombinant IGF-1 has been demonstrated to limit noise-induced hearing loss when applied to the round window; in human studies, IGF-1 administration has been studied in sudden sensorineural hearing loss. Primitive trials have studied IGF-1 (delivered via intratympanic injection or gelatin hydrogels within the middle ear), noting superiority over intratympanic dexamethasone [18]. It is therefore plausible that topical IGF-1 could prevent (or reverse) teprotumumab-related hearing impairment.

When considering teprotumumab for TED, Key et al. suggest counselling all patients on the risk of ototoxicity [19]. Highland et al. additionally advise caution when teprotumumab is used with an additional ototoxic agent and recommend avoiding loud noise exposure above 70 dB [40]. Belinsky et al. recommend audiometry screening before, during, and after completion of teprotumumab [31]. The incorporation of ultrahigh frequency testing should also be considered in those with a normal audiogram and high clinical suspicion [26].

Strengths and Limitations

Original studies included are of high quality, and we address observational studies (real-world) in addition to RCTs. A systematic review of all studies is furthermore incorporated, in addition to reviewing the literature, regarding risk factors, proposed pathophysiology, and potential treatment options. Limitations, however, are present; although we performed a strong database search and included high-quality data, many case reports and case series assisted in the decision for the FDA to add the label warning of hearing impairment. Furthermore, the exact definition of hearing impairment varies within each study, which required us to provide two differing definitions and overall estimates. The true incidence of hearing impairment could be over-reported, as numerous studies do not have consistent audiometry measurements (before, during, or after treatment); similarly, the incidence could be under-reported (as asymptomatic patients may not report symptoms). With the various definitions of hearing impairment and limited follow-up, we could not provide an estimate for recovery of hearing impairment following discontinuation of teprotumumab, nor an estimate of recurrence of hearing impairment upon re-exposure. Concerning the studies analyzed, differences in population, adjustment for confounders, and design can limit overall interpretation. Similarly, observational studies relied on electronic databases, which are prone to misclassification or diagnostic coding, and cannot control for adherence or duration of therapy. Several studies included were funded by the manufacturer of teprotumumab, which may introduce potential funding biases; however, given the limited number of available studies, exclusion of industry-funded trials would reduce the evidence base and limit the interpretability. Such results should be interpreted with caution, and further independent studies are required to confirm these findings. Accordingly, until 2023, the United States was the sole country to use teprotumumab, limiting the external applicability of the results of our study.

## Conclusions

Our meta-analysis addresses hearing impairment related to exposure to teprotumumab, using two different definitions of impairment. The results of this meta-analysis confirm a statistically significant risk amongst both RCTs and observational studies, without heterogeneity. While teprotumumab is associated with significant benefit in the management of TED, the risk of hearing impairment cannot be overstated, and therefore, the potential administration of this medication should be individualized to the candidate most likely to respond and least likely to develop (or be affected by) hearing impairment. Future studies are required to address treatment modalities (such as otic IGF-1 application), risk of recurrence upon re-exposure, and likelihood for recovery after cessation of teprotumumab.
